# Transanal drainage tubes vs metallic stents for acute malignant left-sided bowel obstruction: A systematic review and meta-analysis

**DOI:** 10.1097/MD.0000000000018623

**Published:** 2020-01-10

**Authors:** Jing Xu, Shuai Zhang, Tao Jiang, Yong-Jie Zhao

**Affiliations:** Department of General Surgery, Tianjin Union Medical Center, PR China.

**Keywords:** left-sided malignant bowel obstruction, meta-analysis, metallic stent, systematic review, transanal drainage tube

## Abstract

**Background:**

The surgical management of acute malignant left-sided bowel obstruction is associated with high morbidity and mortality. Recently, transanal drainage tubes (DTs) and metallic stents (MSs) used as a “bridge to surgery” have become widely used decompression methods compared with emergency surgery. This study aims to evaluate the efficacy and safety of DTs and MSs for the decompression of acute left-sided malignant colorectal obstruction.

**Methods:**

All studies were acquired from PubMed, Medline, Embase, CNKI and the Cochrane Library. The data were extracted by two of the coauthors independently and were analyzed with RevMan5.3. Mean differences (MDs), odds ratios (ORs) and 95% confidence intervals (CIs) were calculated. The Cochrane Collaboration's risk of bias tool and the Newcastle-Ottawa scale were used to assess the risk of bias.

**Results:**

Eleven studies, which included three randomized controlled trials (RCTs) and 8 observational studies, were assessed. The methodological quality of the trials ranged from low to moderate. The pooled results of the technical success rate showed that the difference was not statistically significant between the2 devises. The differences in clinical success rate, operative time and complications were statistically significant between MSs and DTs, and MSs were associated with a better clinical success rate, increased operative time and fewer complications. Sensitivity analysis proved the stability of the pooled results, and the publication bias was low.

**Conclusion:**

MS insertion for acute left-sided malignant bowel obstruction is effective and safe with a better technical success rate and with fewer complications than decompression using a DT, and MS insertion can avoid stoma formation. Moreover, MS insertion appears to be a useful treatment strategy for malignant colonic obstruction even if the lesion is located in the right colon. More large-sample, multicenter, high-quality RCTs are needed to verify the outcomes of this meta-analysis.

## 1 Introduction

Colorectal cancer, accounts for 9% of all cancers globally, making it the second most common cancer in women and the third most common in men, is a heterogeneous disease that is caused by the interaction of genetic and environmental factors. Although it is one of the most common cancers worldwide, colorectal cancer would be one of the most curable cancers if it is detected in the early stages.^[1–3]^ Approximately 7% to 29% of patients suffer from colorectal cancer and present with malignant low bowel obstruction.^[4,5]^ Left colorectal cancer accounts for 66% of the total number of cases,^[6]^ and malignant obstruction secondary to left colorectal cancer is a serious disease with rapid progression; the mortality rate associated with untimely treatment is almost 29.27%.^[7,8]^ The traditional treatment choice for malignant obstruction in left colorectal cancer is often staged emergency surgery, including emergency resection of the primary tumor and stoma creation in the proximal colon, followed by stoma closure during the second stage; or creation of a decompression stoma, followed by resection of the primary tumor and stoma closure.^[9,10]^

Staged surgery results in not only secondary surgical trauma to patients but also economic burden and inconveniences in daily life. Clinical data also show that the incidence of complications and mortality from staged emergency surgery are still at a high level.^[11]^

With the continuous development of endoscopic stenting, transanal drainage tubes, the application of potent antibiotics and parenteral nutrition, and the safety and effectiveness of one-stage intestinal anastomosis in patients with colorectal cancer and obstruction are gradually being recognized.^[12,13]^ Clinical data have shown that the success rate of endoscopic intestinal stents and transanal drainage tubes has reached more than 80%, and the incidence of serious complications such as perforation is less than 5%.^[14]^ Compared with emergency surgery, the two methods described above can alleviate obstructive symptoms, avoid emergency surgery, act as a “bridge to surgery”, improve the general condition of patients before surgery, significantly reduce the perioperative mortality of patients, and improve the quality of life of patients.^[15]^

To date, some clinical studies have compared the efficacy and safety of transanal drainage tubes (DTs) and metallic stents (MSs). However, there have been no systematic, quantitative evaluations between the 2 methods. In this article, we included eleven relevant studies to compare the clinical outcomes of DTs and MSs for acute malignant left-sided bowel obstruction to provide some evidence for clinical decision making.

## 2 Materials and methods

The work is reported in line with PRISMA (Preferred Reporting Items for Systematic Reviews and Meta-Analyses) and AMSTAR (Assessing the methodological quality of systematic reviews) guidelines. Ethical approval or patient consent was not required since the present study was a review of previously published literature.

### 2.1 Inclusion criteria for the published studies

#### 2.1.1 Types of studies

We considered all published and unpublished studies covering randomized controlled trials (RCTs) and observational studies, including both retrospective and prospective studies.

#### 2.1.2 Types of participants

All patients were treated for bowel obstruction due to left-sided colorectal cancer. Intestinal obstruction was diagnosed based on clinical findings (severe constipation for > 48 hours, abdominal distension, cramping abdominal pain, nausea and vomiting) and radiological findings (plain radiography and emergent computed tomography of the abdomen). The left side of the colon was defined as the location distal to the splenic flexure. All patients underwent a decompression procedure (with MSs or DTs) within 24 hours after their visit. The exclusion criteria were patients with a variety of severe underlying diseases, benign diseases, right-sided colonic obstruction, partial obstruction that could be managed conservatively, advanced tumors that could not be treated surgically, multiple stenoses or excessive length of stenosis, and emergency surgery.

#### 2.1.3 Types of interventions

All decompression techniques using either an MS or a DT were considered. The exclusion criteria were as follows: insufficient clinical outcome data in studies; comparisons between the MS or DT and others; and reviews or letters.

#### 2.1.4 Types of outcome measures

The primary outcome measures were the technical success rate and clinical success rate. The secondary outcomes included operative time and complications.

#### 2.1.5 Search methods for the identification of studies

Five databases (PubMed, Medline, Embase, CNKI, and the Cochrane Library) were searched using the following keywords: "stent or self-expanding metallic stents or colonic stent or colorectal stent", "surgery or operation", "tube or transanal drainage tube or decompression tube or ileus tube", "random controlled trial" and "intestinal obstruction or large bowel obstruction or colonic obstruction" through December 2018 to collect relevant studies that clinically compared the use of a DT versus an MS for left-sided bowel obstruction. The titles and abstracts of potential related articles identified by the electronic search were reviewed. References from retrieved articles were also assessed to extend the search strategy.

#### 2.1.6 Data collection and quality assessment

Two partners (SZ, JX) independently assessed the titles and abstracts of all the studies screened during the initial search, and they excluded any clearly irrelevant studies using the inclusion criteria. Data were independently extracted using a standard data form for the first author's name, year of publication, sample size, sex, age, intervention, country, tumor location, study design, tumor stage and relevant outcomes. A third partner (TJ) handled any disagreement about the inclusion of a study to reach a consensus. Cochrane Collaboration's risk of bias tool was used for the assessment of quality in the RCT s. Observational studies were assessed by the Newcastle-Ottawa scale, which includes 8 items. A higher overall score indicated a lower risk of bias, and a score of 5 or less (out of 9) corresponded to a high risk of bias.

### 2.2 Statistical analysis

RevMan statistical software 5.3 was used for the meta-analysis. The continuous variables were analyzed as a mean difference (MD) and 95% confidence interval (CI). For the dichotomous outcomes, we calculated the odds ratios (ORs) and 95% CIs. The chi-squared statistic and the I^2^ statistic were used for the test of heterogeneity. A *P* < .05 and an I^2^ > 50% was considered significant heterogeneity, and random-effect models were applied. Otherwise, fixed-effect models were used if there was no significant heterogeneity (*P* ≥ .05, I^2^≤50%). We also performed sensitivity analysis by omitting one study at a time to test the stability of the pooled results. Publication bias is shown by the funnel plot.

## 3 Results

### 3.1 Study identification and inclusion

**Figure 1:**
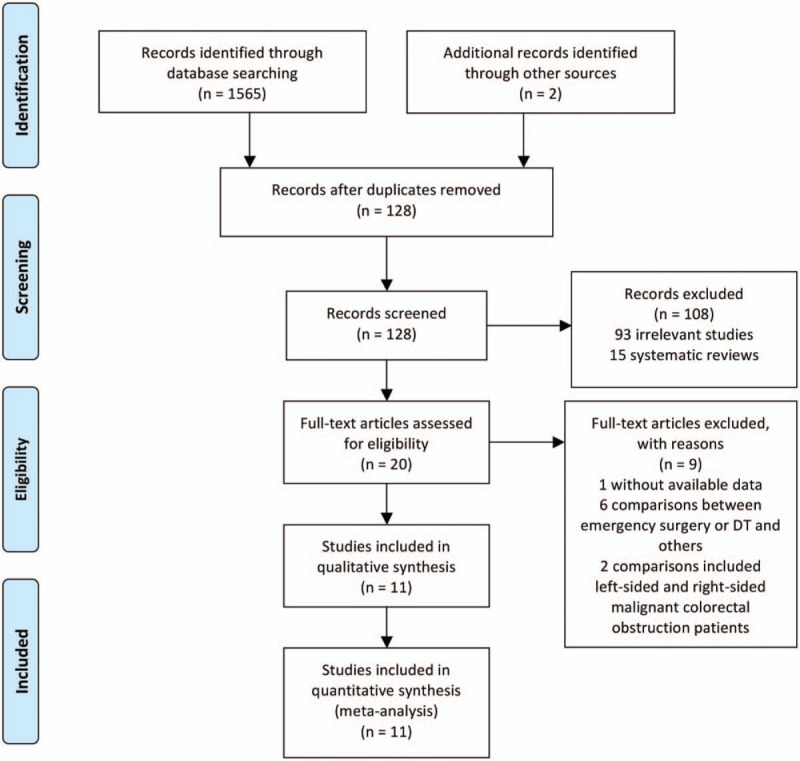
PRISMA flow diagram.

Searches conducted in PubMed, Medline, Embase, CNKI, the Cochrane Library databases and other sources yielded a total of 1567 articles. After removing duplicates, 128 studies remained. Based on the review of the titles and abstracts, 93 irrelevant articles and 15 systematic reviews were excluded. Twenty full-text articles were assessed for eligibility. However, nine articles were excluded based on the previously established exclusion criteria (1 did not have available data, 6 made comparisons between emergency surgery or DTs and others, and 2 made comparisons of left-sided and right-sided malignant colorectal obstruction patients). Finally, eleven trials (3 RCTs and 8 observational studies) were included in this systematic review and meta-analysis. The details of the selection process are listed in Figure 1.

### 3.2 Study characteristics

We assessed 11 studies^[16–26]^ that included 3 RCTs and 8 retrospective studies in this article. The included studies were conducted in 2 countries (China and Japan) from 2010 to 2018 and involved 759 patients (331 patients treated with DTs, 428 patients treated with MSs) aged 43 to 81 years. The most common location of the tumor was in the sigmoid colon (42.4%, n = 291), followed by the rectum (25.6%, n = 176) and descending colon (18.6%, n = 128). The TNM stage was III in 42.6% (n = 60) of the patients, II in 34.8% (n = 49), and IV in 22.7% (n = 32). The Kukos stage was C in 45.4% (n = 113) of the patients, D in 30.1% (n = 75), and B in 24.1% (n = 60). The clinical outcomes of the studies were evaluated mainly based on the technical success rate, clinical success rate, operative time, hospital stay and complications. The detailed information of the included studies is shown in Table 1.

**Table 1:**
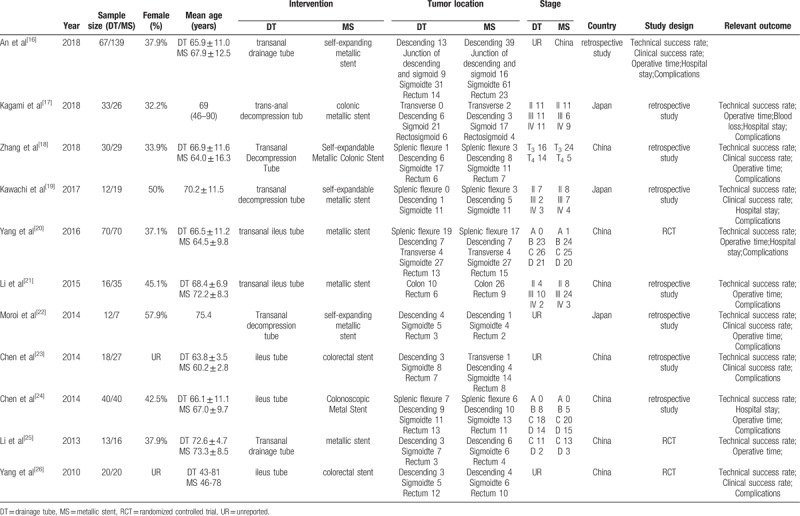
Characteristics of studies included.

### 3.3 Methodological assessment of study quality

**Figure 2:**
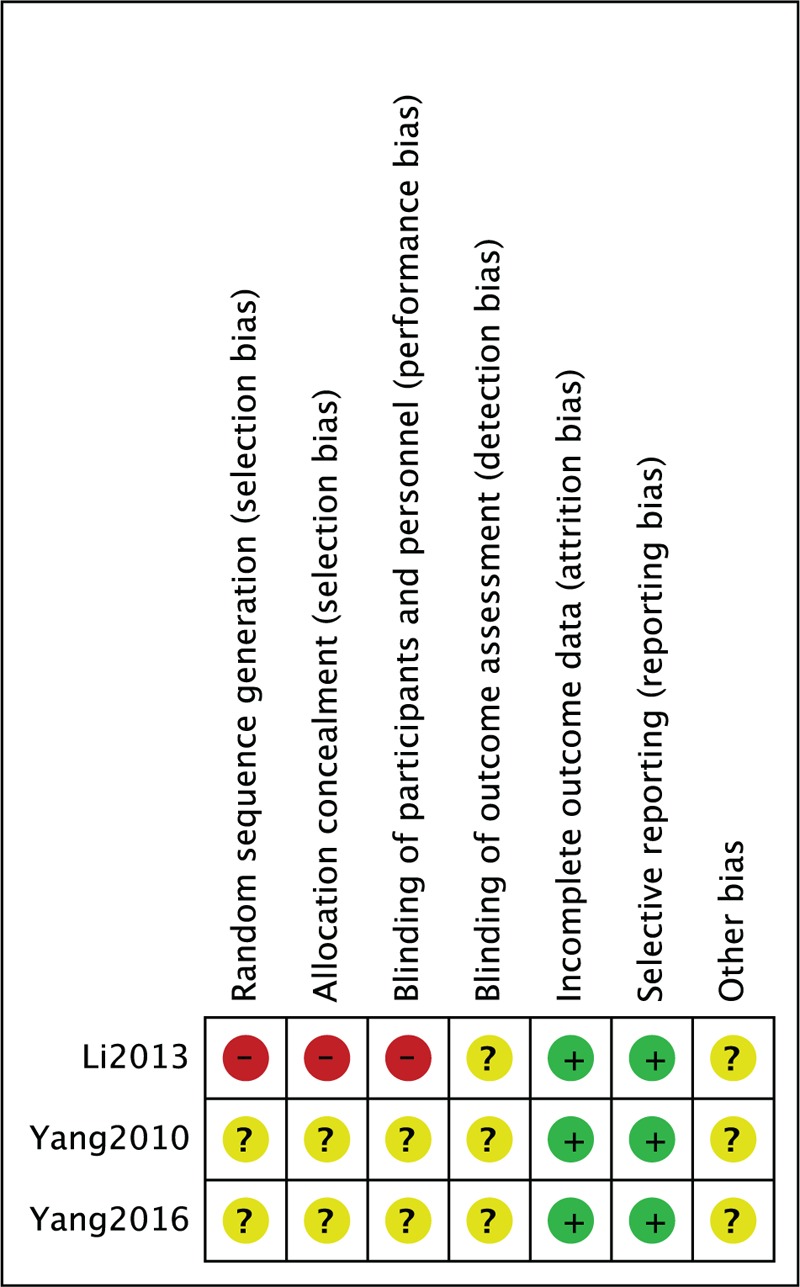
Risk of bias summary: this risk of bias tool incorporates the assessment of randomization (sequence generation and allocation concealment), blinding (participants and outcome assessors), incomplete outcome data, selective outcome reporting, and other risk of bias. The items were judged as “low risk,” “unclear risk,” or “high risk.” Green means “low risk,” red means “high risk,” and yellow means “unclear risk.”

The methodological quality assessment of the 11 included studies is presented in Figure 2 and Table 2. Among the RCTs, Yang study^[20,26]^ did not mention random grouping and thus was considered a low-quality study. Li study^[25]^ described allocation of the patients to either group based on the patient's decision, overall condition, and doctor's evaluation and thus was regarded as a high-risk study. Among the observational studies, the Newcastle-Ottawa scale, including the exposed cohort, the nonexposed cohort, ascertainment of exposure, outcome of interest, comparability, assessment of outcome, length of follow-up and adequacy of follow-up, was used to assess the risk of bias. The score of all 8 studies was 7, indicating a moderate risk of bias.

**Table 2:**
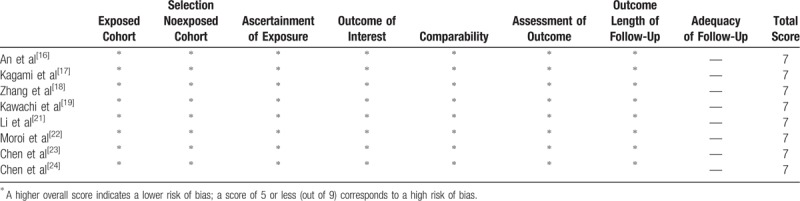
Risk of bias was assessed using the Newcastle-Ottawa Scale.

### 3.4 Comparison of technical success rates between DTs and MSs

**Figure 3:**
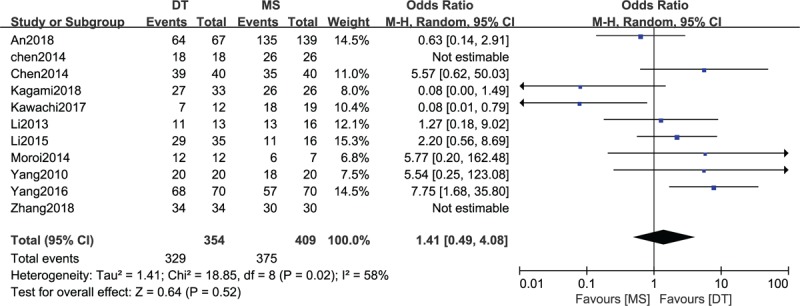
Forest plot of comparison: technical success rate between drainage tube (DT) and metallic stent (MS) for acute left-sided malignant bowel obstruction.

A comparison of the technical success rate between DTs and MSs was conducted among the 11 included studies,^[16–26]^ which included 763 patients (354 patients receiving DTs and 409 patients receiving MSs), as shown in Figure 3. Heterogeneity testing showed that there was moderate heterogeneity among the studies (*P* = .02, I^2^ = 58%), so the random-effect model was used to pool the data from the 11 studies. The pooled results showed that the difference was not statistically significant between the DT group and the MS group (OR = 1.41, 95% CI = 0.49–4.08, *P* = .52).

### 3.5 Comparison of the clinical success rate between DTs and MSs

**Figure 4:**
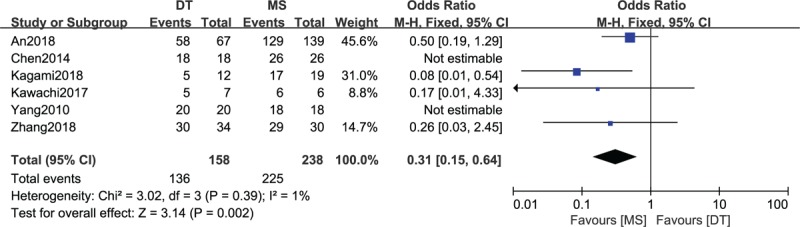
Forest plot of comparison: clinical success rate between drainage tube (DT) and metallic stent (MS) for acute left-sided malignant bowel obstruction.

A comparison of the clinical success rate between DTs and MSs was conducted among the 6 included studies,^[16–19,24,26]^ which enrolled 396 patients (158 patients receiving DTs and 238 patients receiving MSs), as shown in Figure 4. Heterogeneity testing showed that there was low heterogeneity among the studies (*P* = .39, I^2^ = 1%), so the fixed-effect model was used to pool the data for the two groups. The overall estimate showed that the difference was statistically significant between the MS group and the DT group (OR = 0.31, 95% CI = 0.15–0.64, *P* = .002), and the clinical success rate with the use of MSs was better than that of DTs.

### 3.6 Comparison of operative time between DTs and MSs

**Figure 5:**
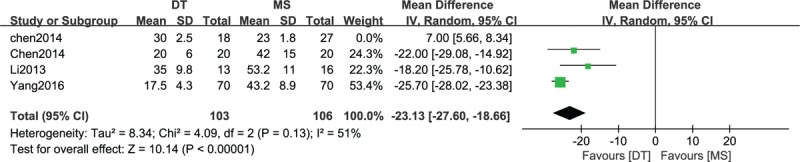
Forest plot of comparison: operative time between drainage tube (DT) and metallic stent (MS) for acute left-sided malignant bowel obstruction.

A comparison of operative time between DTs and MSs was conducted among 4 included studies,^[20,21,23,24]^ which contained 209 patients, as shown in in Figure 5. A heterogeneity test showed that there was moderate heterogeneity among the studies (*P* = .13, I^2^ = 51%), so the random-effect model was used. The overall estimate showed that the difference between the two groups was statistically significant (MD = −23.13, 95% CI = −27.60–18.66, *P* < .00001).

### 3.7 Comparison of complications between DTs and MSs

**Figure 6:**
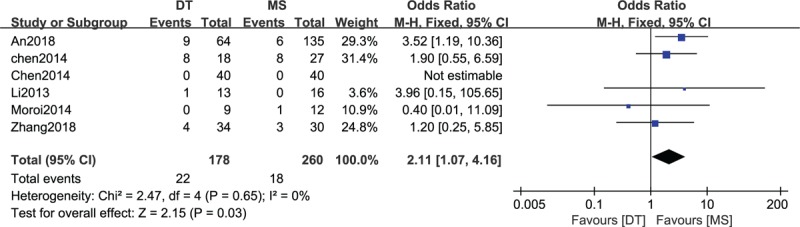
Forest plot of comparison: complications between drainage tube (DT) and metallic stent (MS) for acute left-sided malignant bowel obstruction.

As shown in Figure 6 there were six included studies^[16,18,22–25]^ consisting of 438 patients (178 patients received DTs and 260 patients received MSs) that reported complications. No heterogeneity among the studies (*P* = .65, I^2^ = 0%) was found, so we used the fixed-effect model. The overall estimate indicated that the pooled OR was 2.11 (95% CI = 1.07–4.16, *P* = .03), suggesting that the difference was statistically significant, and the use of MSs was associated with fewer complications than the use of DTs.

### 3.8 Sensitivity analysis and publication bias

**Figure 7:**
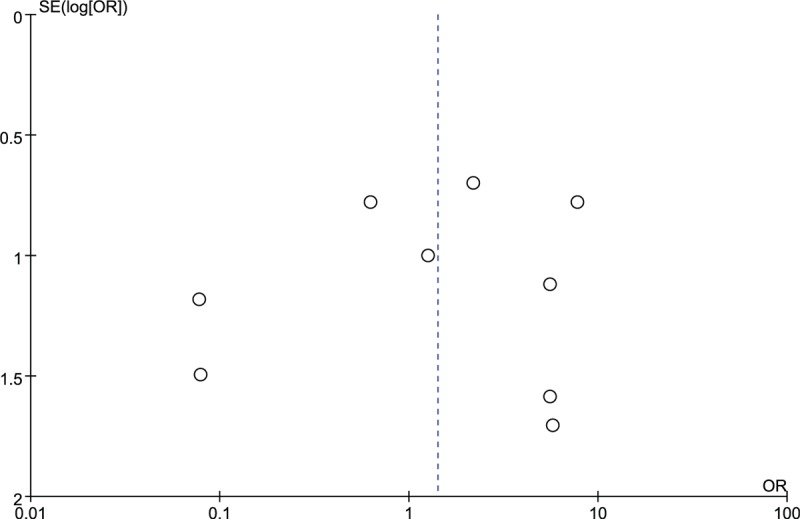
Funnel plot to test for publication bias. Each point represents a separate study for the indicated association. The vertical line represents the mean effects size. OR = odds ratio, SE = standard error.

We performed a sensitivity analysis to assess the stability of the pooled results. Among the majority of studies, the heterogeneity results were not obviously altered after sequentially omitting each study, indicating that our results were statistically reliable. A funnel plot of the included studies is shown in Figure 7. The points in the funnel plot were symmetrically distributed, indicating that the publication bias was low.

## 4 Discussion

The left large intestine is the most common site of cancerous obstruction due to its anatomical, physiological and pathological factors. However, due to the poor general condition of obstructive patients and the insufficient preparation for intestinal cleaning, the incidence and mortality due to surgical complications are high.^[27]^ Traditional surgery usually begins with surgical excision of the fistula followed by selective closure of the stoma. This method causes two instances of trauma to patients, increases the costs for patients and prolongs the recovery period.^[28]^ In recent years, metal stents and intestinal obstruction tubes have been used to alleviate the symptoms of obstruction and avoid emergency surgery. After the obstruction is relieved and the general condition of the patients is improved, a second-stage operation is performed, which reduces the risks associated with the operation and can obviously reduce the mortality rate during the perioperative period as well as improve the quality of life of the patients.^[29]^ By comparing the effects of MSs and DTs on the relief of obstructive symptoms in patients with obstructive colorectal cancer, our results suggested that the difference was not statistically significant between the use of MSs and DTs in terms of the technical success rate. However, different results were discovered in terms of the clinical success rate, operative time and complications, which showed that the use of MSs was associated with a better clinical success rate, increased operative time and fewer complications compared with the use of DTs. This is the first meta-analysis to compare the clinical outcomes of DTs and MSs for acute malignant left-sided bowel obstruction and contribute to provide some evidence for clinical decision making.

During endoscopic decompression treatment, the technical success rate and clinical success rate were 91.7% and 94.5% in the MS group and 92.9% and 86.1% in the DT group, respectively. The clinical remission rate in the MS group was slightly higher than that in the DT group. The reasons may include the following: the stent guide wire is fine and easily passes through tumors; and when the stent is placed, the guide wire easily transmits forces so that the stent can quickly pass through the front of the tumor.

Effective dilatation of the passage and adequate support of the stent made it easy for the proximal feces to pass through the obstruction, thus effectively alleviating the obstruction in the MS group.

This meta-analysis showed fewer complications associated with MSs (18/260, 6.9%) than with DTs (22/178, 12.4%). Perforation occurred in 1 (9.1%) patient 3 days after DT placement, and no severe complications occurred in the MS group in Li's study.^[21]^ Emergency surgery showed that the perforation was due to the long period of compression on the tumor by the balloon. Only 1 case of perforation due to guide wire insertion (left side) was noted, and other complications, such as migration and bleeding, were not observed in Moroi study.^[22]^ In Kagami study,^[17]^ 6 (18.2%) of 33 patients treated with DTs had clinical failure in the form of intestinal perforation, stent migration, or incomplete decompression (3 (9.1%) patients, 2 (6.1%) patients, and 1 (3.0%) patient, respectively). They also reported that surgical site infection occurred in 4 (12.1%) patients in the DT group and 2 (7.7%) patients in the MS group. Both the guide wire and the tube may have stimulated the intestinal wall and induced bleeding or even perforation. Recent studies^[30,31]^ have demonstrated technical improvements in MS insertion with decreased perforation rates reaching 0%. Possible reasons for this are as follows: the guide wire was handled gently, an MS with a low axial force (Niti-S) was used in the majority of patients, and an MS of an appropriate length was selected based on our concept of avoiding contact with the normal colonic mucosa.^[15]^

Tube or stent migration is often caused by long placement, poor placement or tumor growth. The complications of MSs are usually more complex than those of DTs. For example, when stent displacement or tumors continue to grow toward both ends, only new stents can alleviate symptoms. While transanal obstruction tubes are easier to adjust, transanal obstruction tubes can be removed at any time, and MSs must be removed intraoperatively. Therefore, the main method for prevent displacement is to select a stent with a suitable aperture and length. For Kawachi study,^[19]^ two cases of anastomosis leakage were observed in the MS group, but they were controlled with conservative therapy. In Yang study,^[20]^ an incision infection occurred in 5 patients (5/58), obstruction recurrence occurred in 5 patients (5/58) in the MS group, incision infection occurred in 4 patients (4/54), and recurrence occurred in 3 patients (3/54) in the DT group. Incision infection (13.79% in the MS group vs 27.27% in the MT group) and anastomotic leakage (3.45% in the MS group vs 18.18% in the MT group) were reported in Li study.^[21]^ Chen et al^[23]^ reported that surgical site infection occurred in 2 (7.9%) patients in the DT group and two (5%) patients in the MS group. The results described above showed that the incidence of selective surgical infection in the MS group was lower than that in the DT group. The effect of intestinal decompression was better in the stent group, resulting in fewer infections in the surgical area. We believe that early feeding and improvements in intestinal flora and function may also be reasons for these findings.

The stoma rate is related to the degree of intestinal edema. In Takeyama study,^[32]^ significantly greater resolution of histopathologic edema was achieved after placement of MSs than after placement of DTs. By comparing the stoma rate (5.3%, 50.0%, and 56.0%) after MS, DT and emergency surgeries in 56 patients, respectively, the lowest rate was found in the MS group in Kawachi study.^[19]^ In Zhang study,^[18]^ the stoma rate was 20.0% in the DT group and 10.3% in the MS group. These results showed that MSs have the lowest stoma rate by improving intestinal histopathological edema. At the same time, the length of hospital stay was reported in our included studies,^[17,19,20,23]^ and the stent group also showed obvious advantages in terms of the length of hospital stay after surgery. In addition, Matsuda et al^[15]^ compared the endoscopic procedure-related total medical expenses of each devise, and the medical cost of MSs was relatively higher than that of DTs. The benefits of using MSs, including patients’ comfort (tube free), and decompression efficacy, may compensate for the increased expense.

Also, during our pooled results, Kagami et al reported all patients treated with MSs had higher rates of solid food intake and temporary discharge prior to surgery compared with patients treated with DTs. It was advantageous that all patients treated with MSs were able to initiate solid food intake and were able to be discharged from the hospital for a short time when compared to patients treated with DTs. Additionally, surgeries performed after MSs had more complete pathologic staging in terms of more resected lymph nodes.^[17]^ In addition, compared with DT, MS has the advantage of not needing irrigation, no discomfort or pain, no foul odor, and evaluation of the proximal colon in Kawachi study.^[19]^

Some limitations of this study should be noted. First, the small sample size might have affected the significant differences observed between the two devises. Second, MS insertion is currently considered to be a safe and effective alternative modality for decompression, especially in Western countries. DT insertion is only used in limited areas, including Asia. This regional difference may add to the clinical heterogeneity. Three, the proper indications for each procedure (MSs and DTs) are not exactly the same. MS insertion can be performed relatively easily not only for left-sided low bowel obstruction but also for right-sided low bowel obstruction.^[22]^ We only included the left-sided low bowel obstruction patients to decrease the clinical bias. Fourth, and last but not least, the included studies were mostly observational studies and not RCTs, and they largely relied on retrospectively collected data, resulting in a high risk of selection bias. More large-sample, multicenter, high-quality, randomized controlled trials are needed to verify the outcomes of this meta-analysis.

## 5 Conclusions

MS insertion as a “bridge to surgery” strategy for acute malignant left-sided bowel obstruction is effective and safe with a better technical success rate and with fewer complications than decompression using a DT, and MS insertion can avoid stoma formation. Moreover, MS insertion appears to be a useful treatment strategy for acute malignant colonic obstruction even if the lesion is located in the right colon. In light of the heterogeneity and retrospective design, whether these conclusions are applicable should be further determined in future long-term studies.

## Author contributions

**Conceptualization:** Shuai Zhang, Yong-Jie Zhao.

**Data curation:** Shuai Zhang, Yong-Jie Zhao.

**Formal analysis:** Shuai Zhang, Yong-Jie Zhao.

**Funding acquisition:** Tao Jiang, Yong-Jie Zhao.

**Investigation:** Jing Xu.

**Methodology:** Shuai Zhang.

**Project administration:** Jing Xu, Tao Jiang.

**Software:** Shuai Zhang.

**Supervision:** Jing Xu, Shuai Zhang.

**Validation:** Jing Xu, Shuai Zhang.

**Visualization:** Jing Xu, Shuai Zhang.

**Writing – original draft:** Shuai Zhang.

**Writing – review & editing:** Tao Jiang, Yong-Jie Zhao.
